# Chaos in Opinion-Driven Disease Dynamics

**DOI:** 10.3390/e26040298

**Published:** 2024-03-28

**Authors:** Thomas Götz, Tyll Krüger, Karol Niedzielewski, Radomir Pestow, Moritz Schäfer, Jan Schneider

**Affiliations:** 1 Mathematical Institute, University of Koblenz, 56070 Koblenz, Germany; goetz@uni-koblenz.de (T.G.); pestow@uni-koblenz.de (R.P.); moritzschaefer@uni-koblenz.de (M.S.); 2 Faculty of Information and Communication Technology, Wrocław University of Science and Technology, 50-372 Wrocław, Poland; tyll.krueger@pwr.edu.pl; 3 Interdisciplinary Centre for Mathematical and Computational Modelling (ICM), University of Warsaw, 00-927 Warsaw, Poland; 4 Faculty of Management, Wroclaw University of Science and Technology, 50-371 Wrocław, Poland; jan.schneider@pwr.edu.pl

**Keywords:** epidemiology, sociophysics, disease dynamics, opinion dynamics, SIS-model, *q*-voter model, chaos

## Abstract

During the COVID-19 pandemic, it became evident that the effectiveness of applying intervention measures is significantly influenced by societal acceptance, which, in turn, is affected by the processes of opinion formation. This article explores one among the many possibilities of coupled opinion–epidemic systems. The findings reveal either intricate periodic patterns or chaotic dynamics, leading to substantial fluctuations in opinion distribution and, consequently, significant variations in the total number of infections over time. Interestingly, the model exhibits a protective pattern.

## 1. Introduction

Understanding the complex dynamics of opinion formation in a society is a challenging cross-disciplinary task where expertise and knowledge from many different fields are required. Mathematical models of opinion dynamics can provide quantitative and qualitative insights into the set of parameters and variables that shape and determine the dynamics and structure of opinions. Thanks to pioneers like Serge Galam in modeling social phenomena with methods borrowed and inspired by statistical physics, whole new sub-fields of research like sociophysics have been established [1] and triggered subsequently work. Meanwhile, there have been a large variety of models designed like the Galam Models [2], Voter model [3], Sznajd model [4], threshold models [5] and bounded confidence models [6], to name just a few.

Mathematical models for the epidemic dynamics of infectious diseases have existed for more than two hundred years, starting with the work of Bernoulli in 1766 on smallpox inoculation [7] and continuing—after a long pause—with Ross and Hudson on Malaria at the beginning of the last century [8].

However, until the COVID-19 pandemics, there was relative little interest among the epidemiological modeling community to incorporate aspects of opinion dynamics into epidemic models. Some notable exceptions are [9,10,11,12,13,14,15,16]. During the COVID-19 pandemics, it became evident that the efficiency of the implementation of so-called Public Health and Social Measures (PHSMs) depend crucially on the level of acceptance in a society and is, therefore, impacted by opinion formation processes. Furthermore, in most countries of the Western hemisphere, we have seen strong polarization dynamics within society and deep digression on what is “the right thing to do” to fight the pandemic. These processes shaped also the attitude toward COVID-19 vaccination and were causal for the large number of deaths in autumn 2021 due to insufficient vaccine uptake in the elderly population in eastern European countries like Poland and Bulgaria (for major works on coupled opinion–epidemic systems since 2008 see [9,10,11,12,13,14,15,16,17,18,19,20,21,22,23,24,25,26,27,28,29,30,31]).

In this article, we study one of the many possibilities of coupled opinion–epidemic systems. The opinion dynamics we consider are a slight generalization of the so-called *q*-voter model (a kind of nonlinear voter process which favors the most prevalent opinion). The number of possible opinions is large and represents a continuum in our setting. For the epidemic dynamics, we consider a simple SIS system. Opinions have a direct impact on the likelihood of becoming infected (one possible interpretation could be to associate opinions with the frequency of mask wearing) and, therefore, cause heterogeneity in the relative share of infections among the different opinion carriers. In the other direction, an infection increases the likelihood of changing an opinion (more precisely, the likelihood of an infected individual to change their opinion is proportional to the the relative proportion of infected of their opinion among all infected). It is easy to show that the coupled system has no stationary solutions in the general case (Section 2.5) despite the fact that in the decoupled system both—the opinion and the epidemic dynamics—converge to stable equilibria.

It is a widespread belief that pure opinion formation systems are unlikely to show chaotic behavior [32], although there are examples of chaos even in simple deterministic opinion models [33]. We observe for the system presented in this article either complex periodic patterns or chaotic dynamics with large fluctuations in the distribution of the opinions causing substantial variations over time in the total number of infected. It has been known that differences in the perceived risk can have dramatic impact on epidemic dynamics [9], and coupled opinion–epidemic systems ‘can exhibit dynamics that do not occur when the two subsystems are isolated from one another’ [17]. For instance, previous works presented the emergence of periodic dynamics in opinion–epidemic models in which behavior dynamics induced an instability of the endemic equilibrium through a supercritical Hopf bifurcation—an effect leading to large oscillations [15,16]. Furthermore, a recent study presented chaotic behavior in a coupled model induced by delayed response of behavior to epidemic variables [31]. Our opinion dynamics and the coupling to the epidemic dynamics differs from the above-mentioned studies and does not involve delayed response or periodic external triggers like seasonality to induce non-stationary dynamics.

## 2. Materials and Methods—Model Description

The examined dynamical system consists of two coupled dynamical systems: an epidemiological system and an opinion formation system.

### 2.1. Epidemiological System

In the following, we consider infectious disease dynamics under the impact of opinions that affect the likelihood of becoming infected in a closed population of size *N*. Opinions are described by a one-dimensional continuous variable x∈[0,1]. A zero value reflects the point of view in opposition or polarization to the opinion with value 1. Values between 0 and 1 reflect non-extreme opinions. Each individual is required to have one opinion *x*. Let S(t,x) and Z(t,x) denote the number of susceptible or infectious individuals with opinion *x* at time *t*. A straightforward extension of the classical Kermack–McKendrick SIS model [34] leads to the system

(1a) $$\begin{array}{cc} \frac{d}{dt}S\left(t,x\right) & =-\frac{\beta (x)}{N}S\left(t,x\right)\int_{0}^{1}Z\left(t,y\right)\;dy+\gamma Z\left(t,x\right)\;, \end{array}$$

(1b) $$\begin{array}{cc} \frac{d}{dt}Z\left(t,x\right) & =\frac{\beta (x)}{N}S\left(t,x\right)\int_{0}^{1}Z\left(t,y\right)\;dy-\gamma Z\left(t,x\right)\;. \end{array}$$

Here, β(x)>0 denotes an opinion-dependent transmission rate. As a prototypical example, one may consider a linear dependence

(1c) $$\beta \left(x\right)=\beta^{0}+\left(\beta^{1}-\beta^{0}\right)x\;,$$

where $\beta^{0}$ and $\beta^{1}$ denote the transmission rates for the two extreme opinions x=0 and x=1. However, it is advised that $\beta^{0}\neq \beta^{1}$ to observe dynamics diverging from standard SIS model. The recovery rate γ>0 is assumed to be independent of the opinion *x*.

### 2.2. Opinion Formation System

To describe the opinion dynamics, we employ a modification of the *q*-voter model [3]. The classical *q*-voter model (q>1) for a population with just two mutually exclusive opinions reads as

(2) $$v^{\prime }=\alpha \left[\left(1-v\right)v^{q}-vs.{\left(1-v\right)}^{q}\right]=\alpha vs.\left(1-v\right)\left[v^{q-1}-{\left(1-v\right)}^{q-1}\right]\;,$$

where *v* denotes the fraction of individuals having one opinion and 1−v denotes the remaining individuals having the alternative opinion. A change of opinion occurs, if an individuals ”meets” *q* individuals of the other opinion. Then, with rate α, the individuals flips to the other opinion. It is required that α>0, since individuals are attracted to the opinion of others. It is obvious that the simple *q*-model allows for two opinion-polarized equilibria v=0 and v=1 and a balanced equilibrium $v=\frac{1}{2}$. The two polarized equilibria are asymptotically stable, whereas the balanced equilibrium is unstable. To see this, we remark that v=0, v=1 and v=1/2 are the roots of the right-hand side *f*(*v*) of the above ODE. Computing the derivative at these roots, we observe, that $f^{\prime }$(0) =$f^{\prime }$(1)=−α<0 and $f^{\prime }$(1/2) = (q−1) α ${\left(\frac{1}{2}\right)}^{q-1}>0$, showing the local instability or stability of the respective equilibria.

Let *U*(t,x) =S(t,x) +Z(t,x) denote the total number of individuals having opinion *x*, where x∈[0,1] denotes a continuous range of opinions, e.g., ranging between complete rejection of a non–pharmaceutical intervention (x=0) to full agreement with this intervention (x=1). Then, $N=\int_{0}^{1}U\left(t,x\right)\;dx$ equates to the overall population. When q=2 and there is a continuous spectrum of opinions x∈[0,1], a generalization of the classical *q*–voter model (2) reads

(3) $$\frac{d}{dt}U\left(t,x\right)=\left(aU{\left(t,x\right)}^{2}+\epsilon \right)\int_{0}^{1}U\left(t,y\right)\;k\left(x,y\right)\;dy-U\left(t,x\right)\int_{0}^{1}\left(aU{\left(t,y\right)}^{2}+\epsilon \right)\;k\left(y,x\right)\;dy\;.$$

Here, the non–negative kernel k(x,y):${\left[0,1\right]}^{2}\to R_{+}$ denotes the confidence (i.e., trust) of individuals of opinion *x* and *y* in each others judgment when interacted. With rate a>0, this interaction leads to a switch in opinion. Typically, we will assume that k(x,y)=ρ(|x−y|)=ρ(r), depending only on the distance r=|x−y| of the two opinions *x* and *y*. It seems natural to assume that ρ is decaying with *r*, i.e., the further apart the two opinions, the less the trust in others judgment. This mechanism is referred to as *Bounded Confidence* (BC) [6] in this work.

It is important to note that the probability of sampling of an individual with specific opinion in the continuous *x* equals zero. Therefore, in the system, the interaction of individuals should be interpreted as the attraction of an individual to a particular opinion rather than “meeting” other individuals.

The individual can change their opinion according to *q*-individuals with rate *a*. The variable ϵ>0 denotes a background rate of opinion change independent of encounters with other individuals. The proportion of individuals who take action is defined by parameters *a* and ϵ with the assumption 0≤a+ϵ≤1. The proportion of individuals who do not take any action equals 1−a−ϵ.

Some mathematical results for this model can be found in the appendix in Propositions A1 and A2.

### 2.3. Opinion–Epidemic Model

In this section, we will use z(t,x) and u(t,x) as a densities of variables Z(t,x) and U(t,x), respectively (i.e., $z\left(t,x\right)=\frac{Z(t,x)}{\int_{0}^{1}U\left(t,x\right)dx}$ and $u\left(t,x\right)=\frac{U(t,x)}{\int_{0}^{1}U\left(t,x\right)dx}$). To couple the opinion dynamics with the infection process, we assume that the rate of changing one’s opinion *x* scales with the number of infected z(t,x) having this opinion. The rational behind this assumption is the following: If an individual of opinion *x* observes a large number of infected having the very same opinion *x*, the more likely it is that the individual will change to another opinion *y*. Furthermore, conversely, if there are only a few infected sharing one’s opinion, then it is less likely for the individual to change that opinion. Scaling all populations with the total population *N*, incorporating the above idea into Equation (3) and combining it with the the *SIS*-model (1), we arrive at

(4a) $$\begin{array}{cc} \frac{d}{dt}z\left(t,x\right) & =\beta \left(x\right)\;\left(u(t,x)-z(t,x)\right)Z\left(t\right)-\gamma z\left(t,x\right)\;, \end{array}$$

(4b) $$\begin{array}{cc} Z(t) & =\int_{0}^{1}z\left(t,y\right)\;dy\;, \end{array}$$

(4c) $$\begin{array}{cc} \beta (x) & =\beta^{0}+\left(\beta^{1}-\beta^{0}\right)x\;, \end{array}$$

(4d) $$\begin{array}{cc} \frac{d}{dt}u\left(t,x\right) & =\frac{1}{Z(t)}\left(\left(au{\left(t,x\right)}^{2}+\epsilon \right)\int_{0}^{1}z\left(t,y\right)\;u\left(t,y\right)\;\rho (|x-y|)\;dy\right. \end{array}$$

(4e) $$\begin{array}{cc} \; & \;\;\left.-u\left(t,x\right)z\left(t,x\right)\int_{0}^{1}\left(au{\left(t,y\right)}^{2}+\epsilon \right)\;\rho (|x-y|)\;dy\right)\;. \end{array}$$

Here, Z(t) denotes the total number of infected.

A simple model for the interaction kernel ρ(r) is the so-called *bounded confidence interval*

(4f) $$\rho (|x-y|)=\left\{\begin{array}{cc} 1 & \mathrm{for}\;|x-y|\leq \tau \\ 0 & \mathrm{else}\;. \end{array}\right.\;,$$

i.e., if the difference between the two opinions *x* and *y* exceeds the bounded confidence threshold τ, no interaction occurs. Some analytic results for the asymptotic dynamics of the pure opinion dynamics are provided in Appendix A and Appendix B.

### 2.4. Discretization of the Opinion Space

To simulate the coupled infection–opinion dynamics (4), we discretize the opinion space [0,1] by *n* discrete opinions $x_{1}<\cdots <x_{n}$. For simplicity, we assume $x_{k}=h\left(k-\frac{1}{2}\right)$ for k=1,⋯n and h=1/n to be equidistantly spaced. Let $z_{i}\left(t\right)=z\left(t,x_{i}\right)$ and $u_{i}\left(t\right)=u\left(t,x_{i}\right)$. Then, the opinion-discretized version of (4) reads as

(5a) $$\begin{array}{cc} z_{i}^{\prime } & =\beta_{i}\;\left(u_{i}-z_{i}\right)Z-\gamma z_{i}\;, \end{array}$$

(5b) $$\begin{array}{cc} u_{i}^{\prime } & =\frac{1}{Z}\left(\left(au_{i}^{2}+\epsilon \right)\sum_{k=1}^{n}z_{k}\;u_{k}\;\rho_{h}(|i-k|)-u_{i}z_{i}\sum_{k=1}^{n}\left(au_{k}^{2}+\epsilon \right)\;\rho_{h}(|i-k|)\right)\;, \end{array}$$

where

(5c) $$\begin{array}{cc} 1-1Z(t) & =\sum_{k=1}^{n}z_{k}\left(t\right)\;, \end{array}$$

(5d) $$\begin{array}{cc} \beta_{i} & =\beta^{0}+\left(\beta^{1}-\beta^{0}\right)x_{i}\;, \end{array}$$

(5e) $$\begin{array}{cc} z_{i}^{\prime } & =\frac{d}{dt}z_{i}\left(t\right)\;, \end{array}$$

(5f) $$\begin{array}{cc} u_{i}^{\prime } & =\frac{d}{dt}u_{i}\left(t\right)\; \end{array}$$

and $\rho_{h}\left(d\right)=\rho \left(hd\right)$ denotes the scaled bounded confidence kernel.

The coupled 2n-dimensional ODE system (5a,b) can be solved by any standard ODE solver, e.g., a classical Runge–Kutta method with adaptive step sizes. For the simulation implementation details, please see Section 2.6.

### 2.5. Some Analytic Results for a Simplified Setting

Consider the opinion-discretized system (5) in the simplified setting $\rho_{h}\equiv 1$ and ϵ=0. We will show in the following that such a system cannot have a stationary solution except for the case when the likelihood of becoming infected does not depend on the opinion (in that case the system is actually decoupled). Non-trivial equilibria for the infected compartments $z_{i}$ are characterized by the equation

$$z_{i}=\frac{\beta_{i}u_{i}Z}{\beta_{i}Z+\gamma }\;.$$

Analogously, the equilibria for the opinion group $u_{i}$ have to satisfy

$$\begin{array}{cc} u_{i}^{2}\sum z_{k}u_{k} & =u_{i}z_{i}\sum_{k}u_{k}^{2}. \end{array}$$

Inserting the above relation for $z_{i}$, yields

$$\begin{array}{cc} \sum_{k}\frac{\beta_{k}u_{k}^{2}}{\beta_{k}Z+\gamma } & =\frac{\beta_{i}}{\beta_{i}Z+\gamma }\sum_{k}u_{k}^{2}. \end{array}$$

Hence, $\beta_{i}/\left(\beta_{i}Z+\gamma \right)$ has to be a constant independent of *i*. This is only possible if $\beta_{i}=\beta$ is a constant independent of *i*.

Assuming $\beta_{i}=\beta$ to be independent of *i*, we define $A=\frac{\beta Z}{\beta Z+\gamma }$ and hence, $z_{i}=Au_{i}$. Therefore,

$$\begin{array}{cc} Z & =\sum_{i}z_{i}=\frac{\beta Z}{\beta Z+\gamma }\sum u_{i} \end{array}$$

and thanks to $\sum_{i}u_{i}=1$, we arrive at 1=β/(βZ+γ), or

$$\begin{array}{cc} Z & =1-\frac{\gamma }{\beta }. \end{array}$$

Feasible solutions for Z<1 can only exist if β>γ, i.e., if the classical epidemiological threshold condition $R_{0}=\frac{\beta }{\gamma }>1$ for the basic reproduction number $R_{0}$ is satisfied. The result is likely to hold for the general case ($\rho_{h}\neq 1$ and ϵ>0) and values of epsilon that are not too large; however, to date, we have been unable to obtain conclusive analytic results in this direction.

### 2.6. Simulation Setup

#### 2.6.1. Software

We run simulations in Julia [35] version 1.9.4. To resolve systems numerically, we use *DynamicalSystems* [36,37] and *OrdinaryDiffEq* [38]. For the postprocessing and analysis, *ChaosTools* [37], *StatsBase*, and *FFTW* [39] packages are used.

#### 2.6.2. Hardware

Computations are performed on a Cray XC40 (Okeanos), which is part of the ICM computing infrastructure. The system is composed of 1084 computing nodes. Each node has 24 Intel Xeon E5-2690 v3 CPU cores with a two-way Hyper Threading (HT) with 2.6 GHz clock frequency.

#### 2.6.3. Simulations

Simulations were conducted in a discretized opinion space *x* with space points *n*. Simulations run for 30,000 time steps with resolution 1. To allow for the system to stabilize its dynamics, the initial 20,000 steps are discarded and only the last 10,000 steps are used for further investigation.

The coupled 2n-dimensional ODE system (5a) is solved using Verner’s “Most Efficient” Runge–Kutta method with order 6(5) [40].

To analyze the system and find parameter sets with chaotic dynamics, we run simulations with parameter sampling in a grid search manner. We decided to alter values of parameters *n*, ϵ, and τ in Equation (4). The parameters *a*, γ, $\beta_{0}$, and $\beta_{1}$ we kept constant. This setup allowed us to vary and evaluate the influence of the number of discretization space points *n*, the proportion of individuals who change their opinion in unstructured manner ϵ, and the bounded confidence interval τ that controls the limits of mixing of a particular opinion in the population. We found these parameters to be the most important for the process. We set *a* to 0.6 to account for the fact that most people are conformist and follow the majority opinion. To make the system over-critical, we set the limits of the function β(x) (i.e., $\beta_{0}$ and $\beta_{1}$) to be above the γ value. This way, the classical epidemiological threshold condition $R_{0}=\frac{\beta }{\gamma }>1$ is satisfied. Values of $\beta_{0}$ and $\beta_{1}$ equal to 0.11 and 0.225, respectively, were chosen to ensure an increase in transmission with increasing *x*. The initial opinion distribution was uniform u(t=0,x)=1.0. The distribution of initially infected was uniform and equal to 0.01 (i.e., z(t=0,x)=0.01).

Fixed parameters and their values used in the grid search are compiled in Table 1. Varied parameters and their range characteristics are compiled in Table 2.

#### 2.6.4. Analysis Methods

Since we observed either periodic and chaotic behavior, we prepared a set of analysis methods that would permit an evaluation regardless of whether the system is periodic or chaotic and provide an insight into dynamics heterogeneity. We used the following methods:

- autocorrelation;
- maximum Lyapunov exponent (MLE) [41,42];
- spectral Shannon; entropy [43,44,45,46];
- standard Shannon entropy [46];
- Poincaré maps;
- Fourier Transform.

Autocorrelation is a standard tool for detecting periodic signals and their frequencies. In the setup, we compute the maximal value of autocorrelation of arbitrarily chosen lags ranging from 150 to 300. Autocorrelation values should be close to 1.0 for the periodic dynamics. Lyapunov exponents measure rates of separation of nearby trajectories in the flow of a dynamical system. In the analysis, we use the maximum Lyapunov exponent (*MLE*) to account for the maximal exponential separation. A positive *MLE* indicates chaotic dynamics. Spectral Shannon entropy is a measure of the heterogeneity in a signal and is computed on the basis of the square of the amplitudes of its Fourier transform. The values are than normalized to 1 and the standard Shannon entropy is computed. The closer the signal is to white noise, i.e., chaotic, the higher the entropy. Standard Shannon entropy is used as a measure of the heterogeneity in opinion dynamics. It is computed based on the densities of opinions in each time step. Then, by obtaining the maximum, minimum, mean, and range length of all entropies (i.e., difference of maximum and minimum), we gain an insight into the diversity of opinions. A Poincaré map is a standard descriptive method used to determine the periodicity in a system. In our work, we plot $u_{i}\left(t\right)$ vs. $u_{i}\left(t+1\right)$ and $z_{i}\left(t\right)$ vs. $z_{i}\left(t+1\right)$ to visualize orbits. When a system is periodic, it has evident closed loops. The Fast Fourier Transform (FFT) is a standard mathematical method for spectral analysis of discrete signals. It is a tool for describing time series by the frequencies and amplitudes of components.

## 3. Results and Discussion

In this section, we evaluate the coupled model dynamics according to their chaotic or periodic nature. We performed 2870 simulations using grid search to investigate the consistency, heterogeneity, variety, and quality of the results. We present the numerical results along with their qualitative and quantitative analyses.

**Figure 1:**
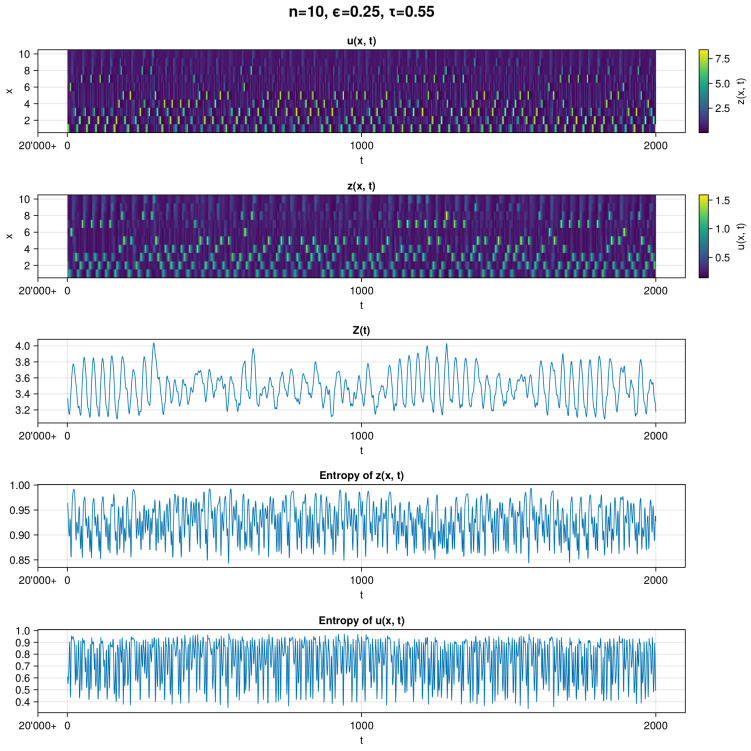
Example of a chaotic timeline for the evolution of the dynamics with parameters n=10, ϵ=0.25, and τ=0.55, where MLE and autocorrelation values indicate chaotic behavior. The timeline begins with 20,000+ time steps and finishes with 20,000 + 2000. We show heatmaps of opinion u(x,t), infected z(x,t), sum of infected Z(t), and entropies of z(x,t) and u(x,t), respectively. Entropies of *z* and *u* are computed with base 10. In each plot, there are fluctuations and irregularities in the data, especially in *u* and entropy of *u*.

**Figure 2:**
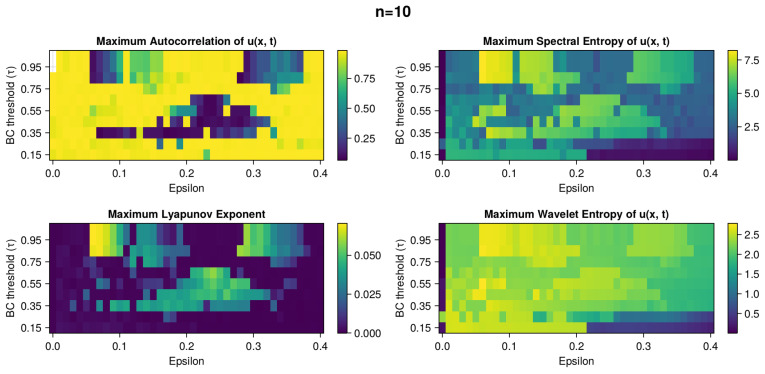
Panel of heatmaps with measures of disorder of dynamics of system with n=10. The results are from the grid search simulations for ϵ and τ parameters that are on the x and y axis, respectively. Maximum Autocorrelation (**top left**), MLE (**bottom left**), Maximum Spectral Entropy (**top right**), and Maximum Wavelet Entropy (**bottom right**) are displayed. In each plot, there are large fluctuations in values. The missing tiles in the Autocorrelation heatmap represent NaN values that mark stationary solutions. The parameter spaces with chaotic simulations are easy to distinguish in the Autocorrelation (low values), MLE (high values) and Spectral Entropy (high values) heatmaps. The low Autocorrelation values, high MLE values, and high Spectral Entropy overlap. This is expected behavior that increases confidence in the existence of chaoticity in these regions. Maximum Wavelet Entropy is an outlier, less consistent with other measures.

In Figure 1, we can see a visualization of the simulation in which the MLE and autocorrelation indicate a chaotic regime. In the top two subplots, we can see visualizations of the raw *u* and *z* data. The absence of clear recurrent patterns is a visual indicator for chaotic behavior (in Figure 2, we see also that for the chosen parameter values, we have positive Lyapunov exponents). This is also apparent for the three bottom subplots, which show aggregate *Z*, entropy of *z*, and entropy of *u*. We can see that *Z* exhibits a deviation in values within ∼20%. Entropy of *u* exhibit a greater range of fluctuations than entropy of *z*. This might be due to the fact that *u* does not translate to the values of *z* as the nonuniform β(x) function is used. Nevertheless, there is little noticeable order in the timelines.

**Figure 3:**
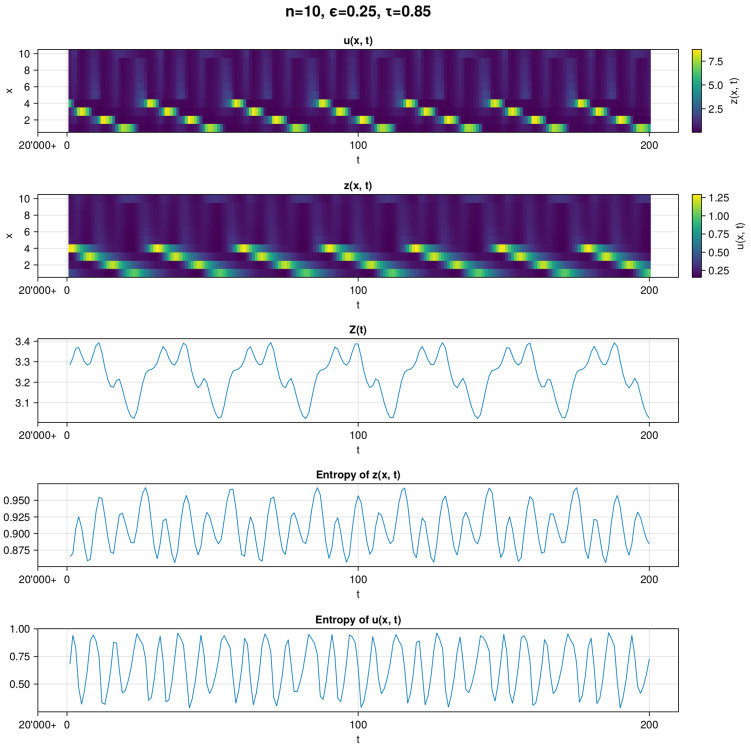
Example of a periodic timeline for the evolution of the dynamics with parameters n=10, ϵ=0.25, and τ=0.85 where MLE and autocorrelation values indicate periodic behavior. The timeline begins with 20,000+ time steps and finishes with 20,000 + 200. We show heatmaps of opinion u(x,t), infected z(x,t), sum of infected Z(t), and entropies of z(x,t) and u(x,t), respectively. Entropies of *z* and *u* are computed with base 10. In each plot, there are fluctuations in the data, especially in *u* and entropy of *u*. One can clearly distinguish periodic behavior in each plot.

In Figure 3, we can see a visualization of a simulation leading to a periodic regime. In the top two subplots, we can see visualizations of the raw *u* and *z* data. A recurrent pattern is readily observable in this example—specifically, *u* and *z* are oscillating in a descending order. Even though the pattern seems simple, it exhibits complex dynamics, observable in the plots of *Z* and both entropies. Similar to the first panel Figure 1, entropy of *u* exhibits a greater range of values than entropy of *z*. However, contrary to the previous panel, we can easily distinguish periodicity in each subplot.

**Figure 4:**
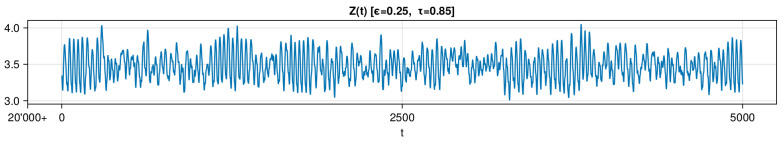
Example of a chaotic timeline for the sum of infected Z(t) from Figure 1. A simulation with parameters n=10,ϵ=0.25, and τ=0.55 with extended time up to 20,000 + 5000 steps is displayed.

In Figure 4, we present extended disordered *Z* dynamics from Figure 1. Notwithstanding, it is evident that the dynamics of the system exhibit pseudo-periodic intervals that are intermittently disrupted by chaotic regimes. While the root cause of this chaotic behavior remains unexplored in this particular study, it serves as a potential hint for further investigations into the underlying pathways leading to chaos.

Up until this point, particular examples of numerical patterns of chaotic and periodic behavior have been presented. Simulations have had easily observable highly nontrivial chaotic behavior and nontrivial periodic behavior. To give a comprehensive perspective on the complexity of dynamics involved in the system, the assessment of disorder in simulations encompassing a diverse array of parameters are illustrated in subsequent figures.

**Figure 5:**
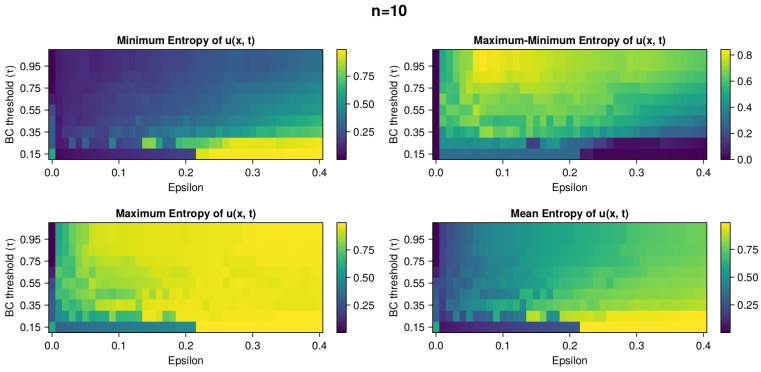
Panel of heatmaps with measures of dispersion in the dynamics of opinion u(x,t) with n=10. The results are from the grid search simulations for ϵ and τ parameters that are on the x and y axis, respectively. Minimum Entropy (**top left**), Maximum Entropy (**bottom left**), difference in Maximum and Minimum Entropy (**top right**), and Mean Entropy (**bottom right**) are displayed. In each plot, there are large fluctuations in values. We can see that the larger the oscillations in the opinion range, the larger the BC threshold. For high epsilon and low BC, we have a very consistent and narrow opinion distribution. The highest oscillations in entropy match with the highest MLE values in Figure 2.

**Figure 6:**
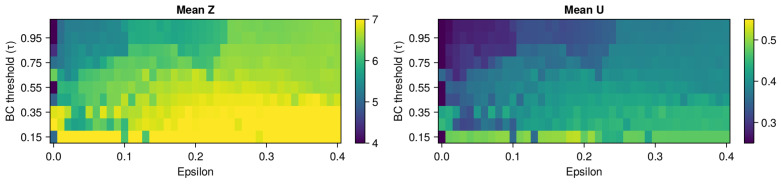
Panel of heatmaps with measures of expected values of the sum of infected Z(t) and opinion u(x,t) with n=10. The results are from the grid search simulations for ϵ and τ parameters that are on the x and y axis, respectively. Mean Z (**left**) and average opinion (**right**) are displayed. In each plot, there are large fluctuations in values. The higher the τ, the lower the mean number of infected and mean opinion. The higher the Epsilon, the higher the mean number of infected and mean opinion. When Epsilon is at its largest value and τ is at it smallest value (bottom right corner), we can see the highest mean number of infected.

The heatmaps in Figure 2, Figure 3, Figure 4, Figure 5 and Figure 6 present the variety of metrics described in Section 2.6.4.

**Figure A1:**
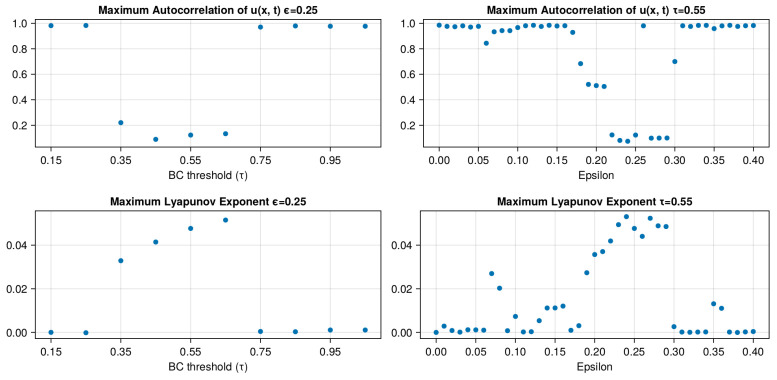
Cross sections along the Epsilon and τ axes of the Autocorrelation and MLE heatmaps in Figure 2. Panel of four scatter plots: Maximum Autocorrelation along the τ axis (**top left**), MLE along the τ axis (**bottom left**), Maximum Autocorrelation along the ϵ axis (**top right**), and Maximum Autocorrelation along the ϵ axis (**bottom right**). We can see large fluctuations in each of the four plots. The low Autocorrelation values correlate with high MLE values, as a comparison of the top and bottom rows shows. This is expected behavior and increases our confidence in the existence of chaoticity in these regions.

The panel in Figure 2 consists of four heatmaps showing the values for Autocorellation, MLE, Spectral Entropy, and Wavelet Entropy. From Figure 1 and Figure 3, we know that *u* displays greater entropy variation than *z*, therefore, we use it to compute autocorrelation and entropy values. The missing tiles in the Autocorrelation heatmap represent the NaN values that mark stationary solutions. The chaotic scenarios are easy to distinguish in the Autocorrelation (low values), MLE (high values), and Spectral Entropy (high values) heatmaps. We can see three nonuniform regions: two in the top and one in the cenere. Overlap of all three is evident with some divergence on the perimeter of chaotic regimes. Convergence of these three measures is a strong computational indicator of chaos in the system. Maximum Wavelet Entropy is an outlier displaying a level of consistency less aligned with the other measures. The chaotic regions in this instance are subtly and delicately outlined. To better visualize the fluctuations and correlation of Autocorrelation and MLE, we include cross-section plots along the x and y axes in the Figure A1.

In Figure 5, there is a panel picture with four heatmaps with statistical properties of entropy of *u*: minimum entropy, maximum entropy, range length of entropy (difference of maximum and minimum value), and mean entropy. We can see that the larger the BC threshold, the larger the oscillations in the opinion range. For high epsilon and low BC, we have a very consistent and narrow opinion distribution. The highest oscillations in entropy match with the highest MLE in Figure 2.

In Figure 6, there are two heatmaps: mean *Z* and mean opinion *x*. The large BC threshold correlates with the low mean of infected and the low mean opinion. Inversely, the lower the ϵ, the lower the mean of the number of infected. This suggests a rational response to epidemics, wherein individuals prioritize self-protection, aligning their opinions with those in favor of a protective regime (lower values of opinion *x*). Notably, when both mechanisms are involved with the strongest influence (largest ϵ smallest τ), we can see the highest mean infected, indicating the worst epidemic outcomes. Surprisingly, there is no visible influence of the chaoticity mode on the mean epidemics results. It remains uncertain whether the mode is irrelevant in general or if the chaotic fluctuations are too subtle to have a noticeable impact on the epidemics.

**Figure 7:**
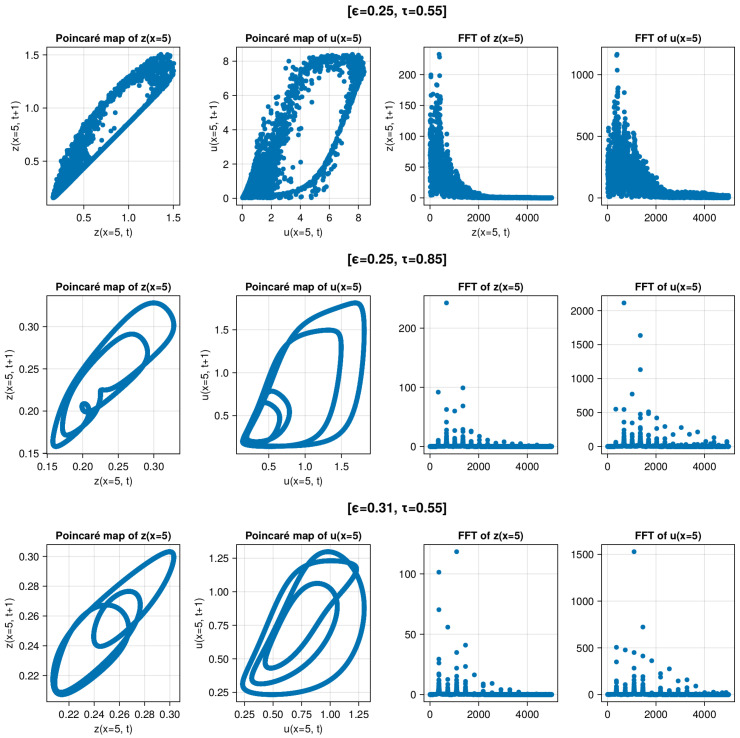
Panel of descriptive statistical analysis plots of three simulations: one chaotic (**top row**) and two periodic (**middle and bottom row**). The top row presents results from a simulation with parameters n=10, ϵ=0.25, and τ=0.55. The middle illustrates a simulation with only the τ parameter changed (0.55 → 0.85). The bottom row of pictures illustrates a simulation with only the ϵ1 parameter changed (0.25 → 0.31). In the first column, there is a Poincaré map of z(x=5). In the second column, there is a Poincaré map of u(x=5). In the third column, there is a Fast Fourier Transform of z(x=5). In the fourth column, there is aFast Fourier Transform of u(x=5). For the sake of clear visualization, we present only absolute amplitudes and half of the frequencies (to avoid a symmetric picture). The Poincaré maps have closed loops in the middle and bottom rows, which differs to the behavior in the top row. The spectral analysis in the top row is highly noisy compares to the analyses below it.

Figure 7 Descriptive statistical analysis of three simulations: one chaotic and two periodic. The chaotic mode simulation is presented in the top row. The middle and bottom rows illustrate simulations with only the τ parameter changed (0.55 → 0.85) and with only the ϵ parameter changed (0.25 → 0.31), respectively. In the two columns on the left-hand side, we have Poincaré maps of z(x=5) and u(x=5). In the two columns on the right-hand side, there are the Fast Fourier Transform results of z(x=5) and u(x=5). For the sake of clear visualization, we present only absolute amplitudes and half of the frequencies (to avoid a symmetric picture). The difference is evident in the panel plots. Poincaré maps have closed smooth loops in the middle and bottom rows, contrary to the behavior in the top row. The spectral analysis in the top row is highly noisy in comparison to those below it. Both periodic and chaotic simulations show highly complex dynamics. Periodic regimes demonstrate nontrivial dynamics even when there is a lack of any noise. We can clearly see that using descriptive statistical methods, we can easily distinguish chaotic from periodic modes and detect the evident noisiness of the chaotic regime.

**Figure 8:**
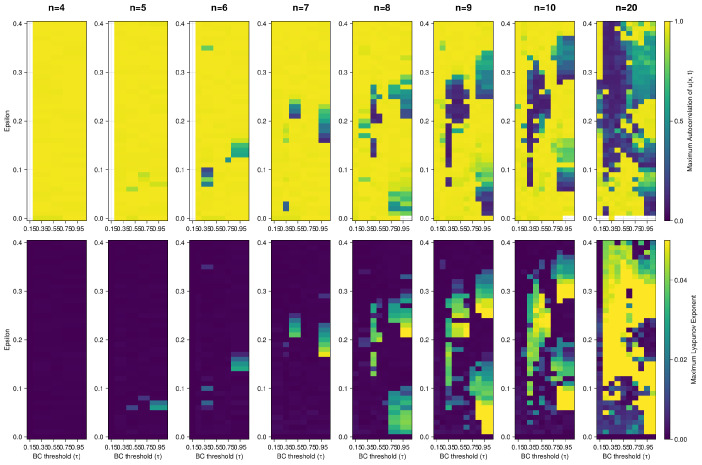
Panel of heatmaps of Autocorrelation and MLE measures according to various discretization resolutions. The first row consists of Autocorrelation heatmaps. The second row consists of MLE. In the columns, we see results for resolutions ranging from n=4 to n=20. The missing tiles in the Autocorrelation heatmaps correspond to NaN values that mark stationary solutions. We can see that the number of confined parameter spaces with chaotic regimes varies between *n* values, and the size of area of unordered dynamics increases with increasing *n*. Chaotic space (positive MLEs) takes up to ∼90% of the total space for n=20.

All the simulations presented up to now had the number of discretization points *n* set to 10. In Figure 8, we present heatmaps for the autocorrelation and MLE measures according to various discretization resolution ranging from 4 to 20 points. The minimal resolution required for the chaotic regime to occur is 5. The missing tiles in autocorrelation heatmap correspond to NaN values that mark stationary solutions. We can see that the number of confined parameter spaces with chaotic regimes varies between *n* values and the size of the parameter space revealing chaotic dynamics increases with increasing *n*. Most of the chaotic parameter regions have unstructured shapes and sizes. The pattern of formation of these areas is noisy and unclear. The positive MLEs marking chaotic space take up to ∼90% of the total space for n=20.

### Discussion

The results show that a minimum of five spatial points is required to observe chaotic behavior. The manifestation of chaotic dynamics is conditioned upon a sufficient number of states and bounded confidence that facilitates the mixing of individuals with at least three different opinions. This outcome aligns with expectations, as, when mixing only two states, there is just one possible opinion transition route (to the opposite opinion). As a result, the opinion formation dynamics are strictly limited and periodicity is enforced.

Subsequently, when the bounded confidence mechanism is deactivated (i.e., τ=1.05), chaotic behavior cases are still observed. This suggests that while bounded confidence may contribute to chaotic dynamics, it is not an obligatory factor for their occurrence. On the contrary, all instances of chaotic dynamics emerge upon the presence of the ϵ>0. This underscores the essential role of opinion change independent of encounters as the mandatory mechanism for chaotic dynamics to develop.

An intriguing aspect of the model is the protective behavior embedded within it. We observe that the strong influence of independent opinion change and BC correlates with worse epidemic outcomes. Interestingly, in scenarios where communication across the population is widespread and uniform, and individuals follow the majority, the average number of infected cases is the lowest. Although we provided numerical evidence for chaotic dynamics only for systems with a finite number n>4 of opinions (up to n=20), we see no reason why chaotic patterns should become absent for large n, which is to say, a continuum of opinions—Figure 8 indicates that the opposite is true. A systematic numerical investigation of the continuous opinion system is challenging, especially when search through the parameter space is required (we observed runtimes of about 2 days for n=20).

## 4. Conclusions and Outlook

This study introduces a new coupled model for opinion and epidemic dynamics. Despite the absence of factors like seasonal effects, delayed response, or contrarians, which are known to trigger chaotic or complex dynamical pattern, our simulations and analyses reveal compelling evidence for both chaotic and complex periodic behavior. This observation is unexpected given that the constituent of the decoupled system is deterministic and exhibits only stationary dynamics and the coupling mechanism is quite simple (infected individuals are just more likely to change their opinion than non-infected).

A closer examination of the chaotic timeline unveils pseudo-periodic intervals disrupted by a noisy signal. This phenomenon might be a hint for a possible route to chaos via intermittency. We did not find any evidence for period-doubling bifurcations.

One implication of chaotic behavior in opinion–epidemics coupled systems is the obvious difficulty in forecasting dynamics beyond a time horizon larger than the inverse of the largest Lyapunov exponent [47]. There are several natural extensions of our system. On the epidemic side, it would be interesting to look at SIRS dynamics. Obviously, many more ways of coupling the opinion and the epidemic dynamics are possible, e.g., the opinion could not only impact the likelihood of becoming infected but also the infectivity of an individual. Finally, there are a large number of opinion models studying the impact of heterogeneity of types of individuals in a society on opinion dynamics, like the contrarians and conformists, and it would be interesting to see how such systems coupled with epidemic dynamics behave.

## Figures and Tables

**Table 1:** Fixed parameters and their values used in a grid search.

**Parameter**	*a*	γ	$\beta_{0}$	$\beta_{1}$
**Value**	0.6	0.1	0.11	0.225

**Table 2:** Varied parameters and their ranges used in a grid search.

**Parameter**	*n*	ϵ	τ
**Initial values**	4	0.0	0.15
**Final value**	10 ^1^	0.4	1.05
**Step size**	1	0.01	0.1

^1^ 20 is appended to the sequence in the end.

## Data Availability

The code used in the research is available under https://doi.org/10.5281/zenodo.10624921 (accessed on 11 March 2024).

## Abbreviations

The following abbreviations are used in this manuscript:

BC	Bounded Confidence
FFT	Fast Fourier Transform
MLE	Maximum Lyapunov Exponent
MDPI	Multidisciplinary Digital Publishing Institute
DOAJ	Directory of Open Access Journals
TLA	Three-Letter Acronym
LD	Linear Dichroism

## Appendix A

**Proposition** **A1.**
*Consider the q-voter model*

$$\begin{array}{cc} \frac{d}{dt}u\left(t,x\right) & =u^{q}\left(t,x\right)\int u\left(t,y\right)\;dy-u\left(t,x\right)\int u^{q}\left(t,y\right)\;dy. \end{array}$$

*Let $\tilde{x}=\mathrm{argmax}\;u\left(t=0,x\right)$. Then $\tilde{x}=\mathrm{argmax}\;u\left(t,x\right)$ for all times t≥0.*

**Proof.** Let the solution *u* in the point $\tilde{x}$ be defined as $\tilde{u}\left(t\right):=u\left(t,\tilde{x}\right)$, and the solution at an arbitrary point $x^{*}$ be defined as $u^{*}\left(t\right):=u\left(t,x^{*}\right)$. We assume $u^{*}\left(0\right)<\tilde{u}\left(0\right)$, so that $\Delta u\left(t\right):=\tilde{u}\left(t\right)-u^{*}\left(t\right)>0$, as well as ${\left(u^{*}\left(t\right)+\Delta u\left(t\right)\right)}^{q}={\tilde{u}}^{q}\left(t\right)$, resulting in the equation $u^{*q}\left(t\right)-{\tilde{u}}^{q}\left(t\right)=-\sum_{k=1}^{q}u^{*q-k}\left(t\right)\Delta u^{k}\left(t\right)$. It thus holds that

$$\begin{array}{cc} {u^{*}}^{\prime }\left(t\right)-{\tilde{u}}^{\prime }\left(t\right) & =\left(u^{*q}\left(t\right)-{\tilde{u}}^{q}\left(t\right)\right)\int u\left(t,y\right)\;dy-\left(u^{*}\left(t\right)-\tilde{u}\left(t\right)\right)\int u^{q}\left(t,y\right)\;dy\\ \; & =-\sum_{k=1}^{q}u^{*q-k}\left(t\right)\Delta u^{k}\left(t\right)\int u\left(t,y\right)dy+\Delta u\left(t\right)\int u^{q}\left(t,y\right)dy \end{array}$$

It holds true that ${u^{*}}^{\prime }\left(t\right)-{\tilde{u}}^{\prime }\left(t\right)<0$ for q=2, since after division by Δu(t)≠0, it holds that

$$\begin{array}{c} \sum_{k=1}^{2}u^{*2-k}\left(t\right)\Delta u^{k-1}\left(t\right)=\sum_{k=1}^{2}u^{*2-k}\left(t\right){\left(u^{*}\left(t\right)-\tilde{u}\left(t\right)\right)}^{k-1}=\tilde{u}\left(t\right)\int u\left(t,y\right)\;dy>\int u^{2}\left(t,y\right)\;dy. \end{array}$$

So, it also holds that ${u^{*}}^{\prime }\left(t\right)<{\tilde{u}}^{\prime }\left(t\right)$ for all q≥2, and thus, $u^{*}\left(t\right)<\tilde{u}\left(t\right)$ for all t≥0. For q≥2, it can be shown by induction that it holds that

$$\begin{array}{c} \sum_{k=1}^{q}u^{*q-k}\left(t\right){\left(u^{*}\left(t\right)-\tilde{u}\left(t\right)\right)}^{k-1}>\int u^{q}\left(t\right)\;dy, \end{array}$$

so that the statement holds for a general q≥2-voter model as well.  □

**Proposition** **A2** (Stability of the equilibrium in the pure 2-voter model)**.** *Regarding the system (3), if ϵ is sufficiently large, i.e., ϵ>a, the uniform equilibrium u(t,x)=1 is stable.*

**Proof.** We consider a perturbation ansatz $u\left(t,x\right)=1+\delta e^{\lambda t}v\left(x\right)$, where $\int_{0}^{1}v\left(x\right)\;dx=0$ for δ≪1. If λ<0, then, the uniform equilibrium u≡1 is stable, otherwise not. Inserting the ansatz into system (3), we obtain

$$\begin{array}{cc} \frac{d}{dt}u\left(t,x\right) & =a\left[\left(u^{2}+\frac{\epsilon }{a}\right)\int_{0}^{1}u\;dy-u\int_{0}^{1}\left(u^{2}+\frac{\epsilon }{a}\right)\;dy\right]\\ \; & =a\left[\left(1+\frac{\epsilon }{a}+2\delta e^{\lambda t}v\left(x\right)+\delta^{2}e^{2\lambda t}v^{2}\left(x\right)\right)\left(1+\delta e^{\lambda t}\int_{0}^{1}v\left(y\right)\;dy\right)\right.\\ \; & \;\left.-\left(1+\delta e^{\lambda t}v\left(x\right)\right)\int_{0}^{1}1+\frac{\epsilon }{a}+2\delta e^{\lambda t}v\left(y\right)+\delta^{2}e^{2\lambda t}v^{2}\left(y\right)\;dy\right]\;. \end{array}$$

Linearizing with respect to δ and using ∫vdy=0, we get

$$\begin{array}{cc} \frac{d}{dt}u\left(t,x\right) & =\delta \left(a-\epsilon \right)e^{\lambda t}vs.+O\left(\delta^{2}\right)\;, \end{array}$$

and thanks to $\frac{d}{dt}u\left(t,x\right)=\delta \lambda e^{\lambda t}v$, we finally arrive at

$$\begin{array}{cc} 1-1\lambda & =\left(a-\epsilon \right)+O\left(\delta \right)\;. \end{array}$$

For ϵ sufficiently large, i.e., ϵ>a, we get λ<0 and, therefore, the stability of the uniform equilibrium u(t,x)=1. As one can see from the above derivation, this result also holds true for the generalization to a q≥2-voter model.  □

The above result shows the local stability of the equilibrium in the 2-voter model. Proving global stability is a different issue, particularly for the integro–differential model. The lack of knowledge of a suitable Lyapunov function is the main obstacle when trying to analyze global stability. Therefore, this issue is still the subject of current research and beyond the scope of this paper.
